# The association of lipid ratios with hyperuricemia in a rural Chinese hypertensive population

**DOI:** 10.1186/s12944-021-01556-z

**Published:** 2021-09-29

**Authors:** Yu Yu, Tian Lan, Dandan Wang, Wangsheng Fang, Yu Tao, Minghui Li, Xiao Huang, Wei Zhou, Tao Wang, Lingjuan Zhu, Huihui Bao, Xiaoshu Cheng

**Affiliations:** 1 Department of Cardiovascular Medicine, The Second Affiliated Hospital of Nanchang University, Nanchang, Jiangxi China; 2 Department of Health Care Management, The Second Affiliated Hospital of Nanchang University, Nanchang, Jiangxi China; 3 Wuyuan County Health Committee, Wuyuan of Jiangxi, Nanchang, China; 4 Center for Prevention and Treatment of Cardiovascular Diseases, Nanchang of Jiangxi, The Second Affiliated Hospital of Nanchang University, Nanchang, China

**Keywords:** Lipid ratios, Hyperuricemia, Hypertension, Lipid ratios, High-density lipoprotein cholesterol, Rural China

## Abstract

**Background:**

Current studies support lipid ratios [the total cholesterol (TC)/high-density lipoprotein cholesterol (HDL-C) ratio; the triglyceride (TG)/HDL-C ratio; the low-density lipoprotein cholesterol (LDL -C)/HDL-C ratio; and non-HDL-C] as reliable indicators of cardiovascular disease, stroke, and diabetes. However, whether lipid ratios could serve as markers for hyperuricemia (HUA) remains unclear due to limited research. This study aimed to explore the association between lipid ratios and HUA in hypertensive patients.

**Methods:**

The data from 14,227 Chinese hypertensive individuals in the study were analyzed. Multiple logistic regression analysis and smooth curve fitting models examined the relationship between lipid ratios and HUA.

**Results:**

The results showed positive associations between the lipid ratios and HUA (all *P* < 0.001). Furthermore, lipid ratios were converted from continuous variables to tertiles. Compared to the lowest tertile, the fully adjusted ORs (95 % CI) of the TC/HDL-C ratio, the TG/HDL-C ratio, the LDL-C/HDL-C ratio, and non-HDL-C in the highest tertile were 1.79 (1.62, 1.99), 2.09 (1.88, 2.32), 1.67 (1.51, 1.86), and 1.93 (1.74, 2.13), respectively (all *P* < 0.001).

**Conclusions:**

The study suggested that high lipid ratios (TC/HDL-C ratio, TG/HDL-C ratio, LDL-C/HDL-C ratio, and non-HDL-C) are associated with HUA in a Chinese hypertensive population. This study’s findings further expand the scope of the application of lipid ratios. These novel and essential results suggest that lipid ratio profiles might be potential and valuable markers for HUA.

**Trial registration:**

No. ChiCTR1800017274. Registered July 20, 2018.

**Supplementary Information:**

The online version contains supplementary material available at 10.1186/s12944-021-01556-z.

## Background

Hyperuricemia (HUA) is a metabolic abnormality syndrome caused by disorders of purine metabolism [1]. Previous studies have suggested that HUA is a close risk factor for metabolic syndrome, chronic kidney disease (CKD), cardiovascular disease (CVD), cardiac death, and all-cause death [2, 3]. Epidemiological studies have shown that the overall prevalence of HUA in China is 13.3 % [4], and the majority of HUA is significantly higher in hypertensive patients [5]. The dramatically increasing prevalence of HUA is a tremendous challenge to public health and constitutes a severe socioeconomic burden [6]. Hence, identifying HUA-related risk factors in the hypertensive population and finding potential valuable indicators could help to improve the management and treatment of chronic diseases.

Previous studies reported that patients with HUA tend to have higher levels of total cholesterol (TC), triglycerides (TGs) and low-density lipoprotein cholesterol (LDL-C), and lower levels of high-density lipoprotein cholesterol (HDL-C) [7, 8]. However, most HUA patients have multiple lipid metabolism disorders [9, 10]; therefore, a single lipid may not be an effective indicator of HUA. Recent emphasis has been placed on lipid ratios as powerful and independent markers of various clinical diseases. For instance, the TC/HDL-C ratio is a well-characterized marker of atherogenic particle burden [11], and previous studies have suggested it is a significant risk factor for CVD [12]. A higher TG/HDL-C ratio and insulin resistance are closely associated [13], and it is also a valuable predictor of CVD [14]. Notably, the LDL-C/HDL-C ratio could better reflect the interaction with lipid metabolism [15] and plays an essential role in early arteriosclerosis and the progression of coronary plaques [16, 17]. In addition, non-HDL-C has been recommended by lipid guidelines as a lipid-lowering target secondary to LDL-C for CVD events [18].

Interestingly, some studies have previously reported the relationship between uric acid (UA) and CVD. However, these studies have different findings, and some studies suggested that UA was positively associated with CVD [19, 20], while others have found a U-shaped relationship [21]. These inconsistent study results have forced us to rethink the specific relationship between UA and CVD. Furthermore, lipid ratios are closely associated with CVD [14, 17]. Exploring the relationship between lipid ratios and HUA may help us better understand the link between UA and CVD. However, there is limited information on the association between these four lipid ratios and HUA in the Chinese population. In addition, more than 1/3 of hypertensive patients have HUA, and these two diseases have a synergistic effect on the occurrence and development of CVD and death [22, 23]. Therefore, the current study explores the independent relationships between the four lipid ratios and HUA in a rural Chinese hypertensive population.

## Methods

### Study design and participants

The study’s data were obtained from the Chinese Hypertension Registry Study (http://www.chictr.org.cn), and details about the purpose, protocol, and outcome of the study have been shown in another study [24]. The inclusion and exclusion procedures of the participants in this study are detailed in Table S1. In short, the study is a large cohort study to explore the prevalence of hypertension in China and the risk factors in hypertensive patients. Hypertension was defined as systolic blood pressure (SBP) ≥ 140 mmHg and/or diastolic blood pressure (DBP) ≥ 90 mmHg measured at baseline or having used antihypertensive drugs to control BP within the normal range [25]. From March to August 2018, the study continuously included 14,268 patients with hypertension from Wuyuan, Jiangxi Province, and all participants signed handwritten informed consent forms. In this study, after excluding 34 non-hypertensive patients and seven patients with missing blood samples, a total of 14,227 hypertensive patients’ data were analyzed in the present study. The clinical research project adhered to the Declaration of Helsinki principles, and it was approved by the ethics committee of Anhui Medical University (No. CH1059).

### Data collection and variable definitions

In the current study, staff with physician qualifications collected venous fasting blood for each study participant, and these blood samples were frozen and stored at Shenzhen Biaojia Biotechnology Laboratory. Laboratory staff analyzed these blood samples using a Beckman automated analyzer (Beckman Coulter, USA). Laboratory indicators included lipids, liver function, renal function, Hcy, and blood glucose. Details of the biochemical indicators are shown in Table 1. After a 10-minute rest, each participant’s blood pressure and heart rate were measured by an electronic sphygmomanometer (Omron HBP-1300, China), evaluated for its effectiveness and accuracy in the previous study [26]. Other covariates were obtained through questionnaires, including age, sex, smoking and drinking status, and disease and medication history. The diagnostic criteria for diabetes were as follows: fasting glucose > 7.0 mmol/L measured at baseline, patients with a previous diagnosis of diabetes, or patients currently taking hypoglycemic drugs [27]. Regarding the definition of HUA, to date, eight guidelines recommend that HUA be diagnosed as serum UA levels > 420 µmol/L (7 mg/dL), regardless of sex, and this is the basis for the definition of HUA in our study [28–30]. Other specific contents of the questionnaire are presented in Table 1.

**Table. 1:** Characteristics of Study Population

Variables*	Total (*n* = 14,227)	non-HUA (*n* = 7,907)	HUA (*n* = 6,320)	*P* value
**Demographics**				
Age, years	63.81 ± 9.36	63.57 ± 8.98	64.10 ± 9.82	< 0.001
Male, %	6716 (47.21)	2624 (33.19)	4092 (64.75)	< 0.001
Current smoking, %	3660 (25.73)	1623 (20.53)	2037 (32.24)	< 0.001
Alcohol use, %	3065 (21.55)	1225 (15.50)	1840 (29.12)	< 0.001
**Comorbidity, %**				
Stroke	983 (6.91)	544 (6.88)	439 (6.95)	0.877
CHD	729 (5.12)	364 (4.60)	365 (5.78)	0.002
Diabetes mellitus	2617 (18.39)	1353 (17.11)	1264 (20.00)	< 0.001
**Medication use, %**				
Antihypertensive drugs	9223 (64.84)	4968 (62.85)	4255 (67.34)	< 0.001
Glucose-lowering drugs	754 (5.30)	417 (5.27)	337 (5.33)	0.877
Lipid-lowering drugs	506 (3.56)	285 (3.60)	221 (3.50)	0.731
**Physical examination**				
BMI, kg/m^2^	23.61 ± 3.74	23.29 ± 3.82	24.01 ± 3.60	< 0.001
SBP, mmHg	148.39 ± 17.86	149.30 ± 17.38	147.25 ± 18.38	< 0.001
DBP, mmHg	88.93 ± 10.75	88.62 ± 10.39	89.31 ± 11.18	< 0.001
**Laboratory results**				
TC, mmol/L	5.16 ± 1.12	5.11 ± 1.08	5.21 ± 1.16	< 0.001
TG, mmol/L	1.81 ± 1.26	1.65 ± 1.06	2.00 ± 1.46	< 0.001
LDL-C, mmol/L	2.98 ± 0.81	2.94 ± 0.79	3.03 ± 0.84	< 0.001
HDL-C, mmol/L	1.57 ± 0.43	1.60 ± 0.43	1.53 ± 0.42	< 0.001
TC/HDL-C ratio	3.44 ± 0.87	3.33 ± 0.81	3.57 ± 0.92	< 0.001
TG/HDL-C ratio	1.32 ± 1.29	1.18 ± 1.06	1.51 ± 1.50	< 0.001
LDL-C/HDL-C ratio	2.01 ± 0.66	1.94 ± 0.62	2.10 ± 0.69	< 0.001
non-HDL-C, mmol/L	3.59 ± 1.00	3.51 ± 0.95	3.68 ± 1.05	< 0.001
Hcy, µmol/L	17.96 ± 11.13	16.29 ± 9.29	20.06 ± 12.77	< 0.001
FBG, mmol/L	6.18 ± 1.61	6.16 ± 1.71	6.21 ± 1.47	0.094
eGFR, ml/min/1.73m^2^	88.18 ± 20.22	94.49 ± 16.18	80.28 ± 21.93	< 0.001

Abbreviations: CHD, coronary heart disease; BMI, body mass index; SBP, systolic blood pressure; DBP, diastolic blood pressure; TC, total cholesterol; TG, triglyceride; LDL-C, low density lipoprotein cholesterol; HDL-C, high density lipoprotein cholesterol; Hcy, homocysteine; FBG, fasting blood glucose; eGFR, estimated glomerular filtration rate

*Data are presented as mean ± standard deviation or median (interquartile range) and numbers (percentage) as appropriate

Note: The TC/HDL-C, TG/HDL-C, and LDL-C/HDL-C ratios were calculated as TC, TG, and LDL-C divided by HDL-C, respectively. Non-HDL-C was calculated as HDL-C subtracted from TC.

### Statistical analysis

The baseline characteristics of the study population are displayed with or without HUA. Continuous variables are usually presented as the mean ± SD, whereas non-normally distributed continuous variables are shown as the median (interquartile range, IQR) and were compared using rank-sum tests. Categorical variables are expressed as numbers (percentages) and were compared by the chi-square test. A logistic regression model was used to analyze the relationship between lipid ratios and HUA. The adjusted variables were selected in the regression analysis model based on the following steps: Step 1, In the multiple linear regression model, the severity of (multiple) collinearities was measured by the variance inflation factor (VIF), with ten as the judgment boundary. When the VIF < 10, there is no multicollinearity and should be adjusted. Step 2, The candidate variables with a *P*-value < 0.05 in the univariate analysis model were included in the multiple regression model [31]. The specific screening steps of the variables are listed in detail in supplementary tables (Tables S2-S5). The trend test was used to evaluate the linear relationship between lipid ratios and HUA when lipid ratios were used as tertiles. In addition, we assessed the goodness of fit of the statistical model. The results showed that the integrity of fit of our statistical model was good (Table S6). Meanwhile, the associations between lipid ratios and HUA were also demonstrated by smoothing curve fitting. Additionally, each cut-off point of the lipid profiles corresponding to HUA (420 µmol/L) was calculated by a recursive algorithm. Sensitivity analysis was used to examine the stability of the associations between lipid ratios and HUA.

All statistical analyses were performed using the statistical packages R (http://www.R-project.org, The R Foundation) and Empower (R) (http://www.empowerstats.com, X&Y Solutions, Inc., Boston, MA). Statistical significance was defined as two-tailed *P* < 0.05.

## Results

### Baseline characteristics of the study participants

According to the inclusion and exclusion criteria, the study enrolled 14,227 hypertensive participants for analysis (mean age: 63.81 ± 9.36 years; 47.21 % males). As shown in Table 1, the participants in this study were divided into two groups according to their HUA status (non-HUA and HUA). Compared with the non-HUA group, participants in the HUA group were older, male. And had higher rates of smoking, drinking, and disease history (including a history of CHD, diabetes, and antihypertensive medication use), higher BMI and DBP values, higher lipid levels (including TC, TGs, LDL-C, TC/HDL-C ratio, TG/HDL-C ratio, LDL-C/HDL-C ratio, non-HDL-C) and higher levels of Hcy and FBG, but lower levels of SBP, HDL-C and eGFR (all *P* < 0.05).

### The association of lipid ratios with HUA

**Fig. 1:**
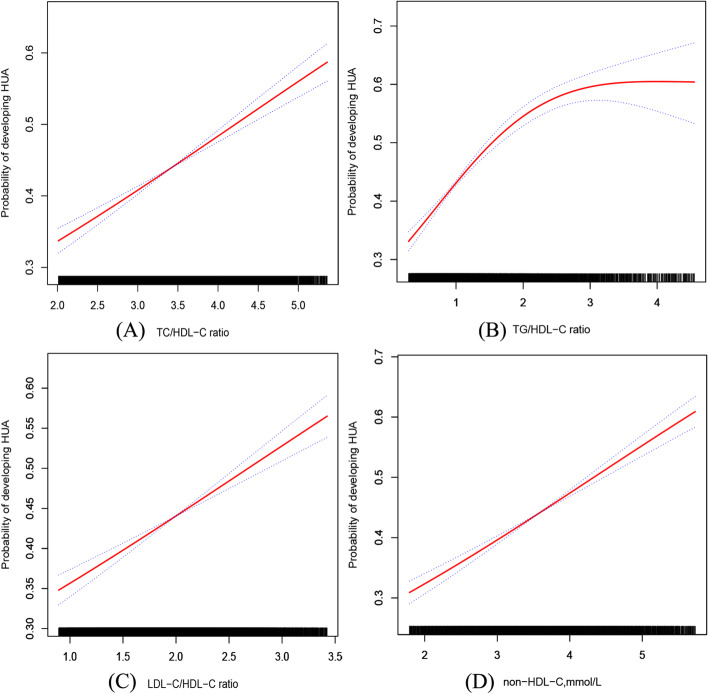
The association between TC/HDL-C (**A**), TG/HDL-C (**B**), LDL-C/HDL-C ratio (**C**), or non-HDL-C (**D**) and HUA. Adjusted for age, sex, BMI, SBP, DBP, current smoking, alcohol use, eGFR, Hcy, diabetes mellitus, antihypertensive drugs, and lipid-lowering drugs

Figure 1 shows the results of the smooth curves between lipid ratios and HUA. Figure 1 A, B, C, and D show that higher TC/HDL-C, TG/HDL-C, and LDL-C/HDL-C ratios and higher non-HDL-C were associated with HUA. Moreover, the cut-off points for the lipid profiles corresponding to HUA (420 µmol/L) are shown in Fig. S1 and Table S7.

A multiple logistic regression analysis model obtained the specific relationships between lipid ratios and HUA, and the results are shown in Table 2. In the unadjusted and adjusted models, higher lipid ratios were positively associated with HUA (all *P* < 0.001). After adjusting for all variables, an increment of 1 SD in the TC/HDL-C, TG/HDL-C, and LDL-C/HDL-C ratios, and non-HDL-C was associated with more significant ORs (95 % CI) of 1.36 (1.29, 1.43), 1.28 (1.23, 1.33), 1.42 (1.33, 1.51), and 1.35 (1.30, 1.41) for HUA, respectively (all *P* < 0.001). Furthermore, lipid ratios were converted from continuous variables to tertiles. Compared with group T1, the adjusted ORs (95 % CI) of the relationship between the TC/HDL-C, TG/HDL-C, and LDL-C/HDL-C ratios, and non-HDL-C with HUA in the T3 group were 1.79 (1.62, 1.99), 2.09 (1.88, 2.32), 1.67 (1.51, 1.86), and 1.93 (1.74, 2.13), respectively (all *P* < 0.001). All *P* values for trends < 0.001 indicated that the associations of these lipid ratios with HUA were more likely to be linear.

**Table. 2:** Odds ratios (95 % CI) for HUA according to continuous and tertiles of lipid ratios

			Crude model		Adjusted model	
Variables	N	Events, n (%)	OR (95 %CI)	*P* value	OR (95 %CI)	*P* value
**TC/HDL-C ratio** (Per 1 SD increase)	14,227	6320 (44.42 %)	1.37 (1.32, 1.43)	< 0.001	1.35 (1.28, 1.41)	< 0.001
Tertiles of TC/HDL-C ratio						
T1 (< 2.75)	4,742	1795 (37.85 %)	Reference		Reference	
T2 (2.75–4.12)	4,742	2032 (42.85 %)	1.23 (1.13, 1.34)	< 0.001	1.32 (1.20, 1.46)	< 0.001
T3 (≥ 4.12)	4,743	2493 (52.56 %)	1.82 (1.68, 1.97)	< 0.001	1.78 (1.61, 1.97)	< 0.001
*P* for trend			< 0.001		< 0.001	
**TG/HDL-C ratio** (Per 1 SD increase)	14,227	6320 (44.42 %)	1.26 (1.22, 1.30)	< 0.001	1.27 (1.23, 1.32)	< 0.001
Tertiles of TG/HDL-C ratio						
T1 (< 0.62)	4,740	1773 (37.41 %)	Reference		Reference	
T2 (0.62–2.03)	4,744	2002 (42.20 %)	1.22 (1.13, 1.33)	< 0.001	1.30 (1.17, 1.43)	< 0.001
T3 (≥ 2.03)	4,743	2545 (53.66 %)	1.94 (1.79, 2.10)	< 0.001	2.09 (1.88, 2.32)	< 0.001
*P* for trend			< 0.001		< 0.001	
**LDL-C/HDL-C ratio** (Per 1 SD increase)	14,227	6320 (44.42 %)	1.45 (1.38, 1.53)	< 0.001	1.40 (1.32, 1.50)	< 0.001
Tertiles of LDL/HDL-C ratio						
T1 (< 1.49)	4,741	1817 (38.33 %)	Reference		Reference	
T2 (1.49–2.53)	4,743	2040 (43.01 %)	1.21 (1.12, 1.32)	< 0.001	1.31 (1.19, 1.45)	< 0.001
T3 (≥ 2.53)	4,743	2463 (51.93 %)	1.74 (1.60, 1.89)	< 0.001	1.65 (1.49, 1.82)	< 0.001
*P* for trend			< 0.001		< 0.001	
**non-HDL-C** (Per 1 mmol/L increase)	14,227	6320 (44.42 %)	1.19 (1.15, 1.23)	< 0.001	1.33 (1.28, 1.39)	< 0.001
Tertiles of non-HDL-C						
T1 (< 2.81)	4,742	1933 (40.76 %)	Reference		Reference	
T2 (2.81–4.36)	4,732	2020 (42.69 %)	1.08 (1.00, 1.17)	0.0575	1.29 (1.17, 1.42)	< 0.001
T3 (≥ 4.36)	4,753	2367 (49.80 %)	1.44 (1.33, 1.56)	< 0.001	1.89 (1.71, 2.09)	< 0.001
*P* for trend			< 0.001		< 0.001	

Abbreviations: OR, odds ratio; CI, confidence interval; TC, total cholesterol; TG, triglyceride; HDL-C, high-density lipoprotein cholesterol; LDL-C, low-density lipoprotein cholesterol; non-HDL-C, non-high-density lipoprotein cholesterol

Adjusted model: adjusted for age, sex, BMI, SBP, DBP, current smoking, alcohol use, eGFR, Hcy, diabetes mellitus, antihypertensive drugs, and lipid-lowering drugs

### Sensitivity analysis

The robustness of the associations between lipid ratios and HUA was verified by sensitivity analysis. After excluding smokers, alcohol drinkers, those with an eGFR < 30 ml/min/1.73m^2^, diabetic patients, and those on lipid-lowering drugs, the associations of these lipid ratios with HUA were analyzed in different populations (Tables S8-S12). These results indicated that the relationship between lipid ratios and HUA was stable and not affected by confounding factors.

## Discussion

The present study findings offer novel evidence for an independent positive association of lipid ratios with HUA in this large sample of Chinese hypertensive patients. These findings fill a gap in the knowledge in the field of lipid research and UA research.

Increasing evidence suggests that lipid ratios could be a valuable indicator for a variety of diseases. Wang et al. [15] analyzed data from 3,259 patients with hypertension (mean age: 58.78 ± 10.20 years) and found those lipid ratios were positively correlated with reduced eGFR. Wang et al. [32] analyzed data from 10,756 Chinese individuals (mean age: 53.8 years) and found that lipid ratio were positively correlated with CVD risk. Wang et al. [33] analyzed data from 2,944 hypertensive patients (mean age: 57.09 ± 11.29 years); positive associations were found between lipid ratios and diabetes, and the TG/HDL-C ratio was more significantly correlated with diabetes. Therefore, we have sufficient reasons to believe those lipid ratios are adequate indicators of these diseases, including stroke, CVD, diabetes, and CKD. Notably, HUA is closely related to the occurrence of these diseases [3, 29, 34]. Therefore, the prevention and treatment of HUA have great clinical benefits. However, few studies have reported the associations between lipid ratios and HUA, so whether lipid ratios can serve as valuable markers of HUA is not clear. The findings extend the application of lipid ratios and fill in the gaps of previous studies; thus, this study has crucial clinical significance.

As mentioned in the Introduction, some studies have explored the relationship between UA and CVD, but the results were inconsistent. Previous studies have suggested that UA is a risk factor for CVD and cardiovascular death [19, 35], while others have suggested a U-shaped relationship, with higher and lower UA levels being associated with CVD [21]. Similarly, the role of UA as a prooxidant or an antioxidant is controversial in basic research [36]. A recent study suggested that the cut-off point between UA and CVD was lower than the traditional definition of HUA, implying that there may be other mechanisms involved in CVD in addition to urate deposition [37]. Therefore, it is necessary to explore the association between different factors and HUA. This study showed positive relationships between lipid ratios and HUA and a possible positive association between HUA and CVD. In other words, our study results suggested that higher UA levels were a risk factor for CVD.

For the U-shaped relationship between UA and CVD found in previous studies, the underlying mechanism may be that higher UA increases the risk of death by stimulating inflammation and oxidative stress. At the same time, lower UA represents malnutrition and a decrease in antioxidant capacity [38]. Another study from the same population showed that the higher UA level was associated with increased TG and LDL-C values but decreased HDL-C values [8]. We did not find a U-shaped relationship between UA and lipid profiles in our study population. It may be because our study population is a general population, with few patients suffering from malnutrition. As a rural population, the diet usually contains more vegetables and fruits, with specific antioxidant capacities [39]. These factors may be why the low UA level in this study population is not considered a risk factor. Interestingly, previous studies have shown that UA has antioxidant capacities, contributing more than 50 % of the oxidation capacity in the blood [40]. The level of UA in the body may be related to vitamins C and E [41]. Therefore, a higher UA level may be beneficial to the body’s metabolism to some extent. However, more basic studies are needed to explore the specific role of UA in the human body.

In the lipid profile, LDL-C and arteriosclerosis-related diseases are most closely related, and apolipoprotein B (ApoB) is an essential component of LDL-C [18]. A recent study reported that UA and ApoB together had synergistic effects on all-cause death and cardiovascular death. Importantly, dietary intervention could reduce this effect, showing that reducing lipid and UA levels has critical clinical benefits [42]. In addition, another study suggested that insulin-like growth factor 1 (IGF-1) is involved in regulating LDL-C levels, which implies the complexity of lipid metabolism [43]; more studies are needed to explore the mechanism of lipid metabolism in the future. Of note, higher BMI is associated with lipid and UA levels, so that BMI may influence the relationship between lipids and UA [44]. In the future, we will try to explore the relationship between lipids and UA in populations with a normal BMI to reduce BMI. In addition, there are sex differences in the relationship between UA levels and cardiovascular events [45]. Therefore, we also performed a sex-stratified analysis for the relationship between lipid ratios and HUA (Table S13). The results showed sex differences in the relationship between the TC/LDL-C ratio and HUA and between the LDL-C/HDL-C ratio and HUA. The reason behind this phenomenon may be that postmenopausal women have higher TC levels than men of the same age (Table S14). Studies have shown that LDL-C often parallels TC levels, indicating that patients with higher TC levels usually have higher LDL-C levels [46]. It may be why the relationship between the TC/HDL-C ratio and HUA and the relationship between the LDL-C/HDL-C ratio and HUA is more significant in the female population. Of note, the rates of antihypertensive drug use in the HUA group and non-HUA group were 67.34 % and 62.85 %, respectively, and it is due to the study population comes from rural areas. Most patients will not actively choose to go to the hospital for a physical examination if they are not unwell; as a result, many of these patients do not realize that they have hypertension. These patients tended to have higher or even extremely high blood pressure at baseline because they had not taken antihypertensive drugs. We presented the frequency of SBP distribution in our study population as a histogram and table (Fig S2 and Table S15). The results showed that 70 % of the study population had SBP values higher than 140 mmHg, which contributed to the high SBP values in the group.

Meanwhile, we noted that all lipid ratios had similar effects on HUA. Regarding this issue, we think it is mainly related to the following two aspects. On the one hand, previous studies have reported that higher lipid ratio levels were associated with CVD [15, 47]. Thus, we can conclude that the relationship between these lipid ratios and CVD has similar effects. Taken together, we can explain the similarity of these effects in terms of the intrinsic links between diseases. On the other hand, we think it should be explained from the calculation and composition of these lipid ratios. In addition, some studies reported that HDL-C was negatively related to UA [8], which also confirms the similar effect of the relationship between these lipid ratios and HUA. In addition, the smooth curve relationship between the TG/HDL-C ratio and HUA (Fig. 1, Panel B) is different from those in the other figures. The main reason is caused by the distribution of TG (Fig S3 and Fig S4). Guidelines have suggested that TG levels in the general population show a significantly positively skewed distribution [46]. Compared with other lipids, the difference in TG levels among individuals was > 20 % [48].

Some reasonable factors may contribute to the associations between lipid ratios and HUA. The relationship between lipid ratios and HUA may be associated with reduced renal function, inflammation, insulin resistance (IR), lifestyle, and lipid-lowering drugs. Lipids deposited in the intima are phagocytosed by monocyte macrophages to become foam cells, causing renal arteriosclerosis, which results in reduced renal function [49, 50]. Higher UA levels form urate crystals that deposit in the kidneys, resulting in decreased renal function [51, 52]. Thus, lipids and UA may interact through decreased renal function. The relationship between the TC/HDL-C and TG/HDL-C ratios and HUA may also be associated with the CRP-mediated inflammatory response [53, 54]. IR may be involved in the relationship between lipids and HUA through inflammation and endoplasmic reticulum stress [55]. In addition, some researchers have found hyperlipidemia, and HUA patients share the same lifestyle and diet, such as excessive alcohol consumption and high-fat food intake, which also suggests that clinicians should conduct lipid-lowering and UA-lowering treatments simultaneously [56]. Interestingly, Deedwania et al. found that lipid-lowering drugs reduced lipid levels and reduced SUA in patients with CVD [57]. There was a close relationship between lipid ratios and HUA. However, current basic studies cannot entirely explain the mechanism of this relationship, and more basic research is needed in the future.

## Study advantages and limitations

The most important advantage is that the study explores the relationships of lipid ratios with HUA in a Chinese hypertensive population for the first time. However, some limitations should be mentioned. First, since the data of this study were from the baseline population without follow-up, the conclusion cannot confirm the causal relationships between lipid ratios and HUA. Second, the study’s findings may not generalize to other countries and regions because the study population was limited to Chinese hypertensive patients. Third, the issue of population selection bias due to hypertensive factors exists in our study. Some hypertensive patients may choose to take diuretics to control their blood pressure, which may have affected the study results considering that diuretics cause an increase in serum UA levels[58].

On the other hand, some hypertensive patients are susceptible to CVD, and they usually choose to use lipid-lowering drugs to reduce lipid levels and delay the CVD progression [48]. Therefore, the patients’ antihypertensive drugs and lipid-lowering drugs and their classifications are listed in Table S16. Diuretics, atorvastatin, and simvastatin were used in 756 (5.31), 141 (0.99), and 260 (1.83) patients, respectively, and these drugs affect the excretion of UA to varying degrees [59]. These potential selection biases result from hypertension and need to be considered for their possible impact on our results. Fourth, survival bias cannot be ruled out in our study. Our findings were derived from an analysis of data from hypertensive patients who survived the baseline period. However, some patients may have died from CVD due to hypertension, dyslipidemia, or HUA before enrollment in our study. Since our study is cross-sectional and the data of deceased patients could not be reviewed and extracted, it was impossible to evaluate whether these patients impacted our findings. Therefore, our conclusions may not comprehensively reflect the actual relationship between lipid ratios and HUA in hypertensive patients. This bias exists in our study, but it cannot be eliminated because of the nature of cross-sectional studies; cohort studies may be needed in the future.

## Conclusions

In a rural Chinese hypertensive population, increased lipid ratios (TC/HDL-C, TG/HDL-C, LDL-C/HDL-C ratios, and non-HDL-C) were associated with HUA. These novel and essential findings suggested that the occurrence of HUA may be related to changes in multiple lipids. Clinically, abnormal lipid metabolism and HUA usually coexist in hypertensive patients, contributing to CVD in patients with hypertension. For clinicians, it is easier to obtain lipid ratios and UA, and it is suitable for clinical promotion and application. Comprehensive management of lipid and UA levels in hypertensive patients may help reduce CVD events and achieve more significant clinical benefits.

## Supplementary information

**Additional file 1**

## Data Availability

The datasets used and/or analyzed during the current study are available from the corresponding author on reasonable request.

## Abbreviations

CKD: Chronic kidney disease
CVD: Cardiovascular disease
T2DM: Type 2 diabetes mellitus
BMI: Body mass index
SBP: Systolic blood pressure
DBP: Diastolic blood pressure
TC: Total cholesterol
TG: Triglyceride
LDL-C: Low-density lipoprotein cholesterol
HDL-C: High-density lipoprotein cholesterol
HUA: Hyperuricemia
UA: Uric acid
Hcy: Homocysteine
FBG: Fasting blood glucose
eGFR: estimated glomerular filtration rate
