# Supplementary material for: The association of lipid ratios with hyperuricemia in a rural Chinese hypertensive population
Source: Lipids Health Dis. 2021 Sep 29;20:121. doi: 10.1186/s12944-021-01556-z (PMC8482679; doi:10.1186/s12944-021-01556-z)
Supplement: Supplementary file 1 — Additional file 1 [file 12944_2021_1556_MOESM1_ESM.docx]

| **Table S1.** The study inclusion and exclusion criteria |
| --- |
| Inclusion criteria |
| 1. 18 years of age or older |
| 1. hypertension defined as office systolic blood pressure (SBP) values ≥140 mmHg and/or diastolic BP (DBP) values ≥90 mmHg, self-reported history of hypertension, or the use of antihypertensive drug(s) at baseline |
| 1. signed informed consent |
| Exclusion criteria |
| 1. psychological or nervous system impairment resulting in an inability to demonstrate informed consent |
| 1. unable to be followed up according to the study protocol, or plans to relocate in the near future |
| 1. patients who were not suitable for inclusion |

**Table S2. Screening for variables in the relationship between TC/HDL-C ratio and HUA**

| Step 1-Collinearity screening | |
| --- | --- |
|  | Step 1 (VIF) |
| **TC/HDL-C ratio** | 1.1 |
| Sex | 1.6 |
| Age | 1.9 |
| BMI | 1.2 |
| SBP | 1.7 |
| DBP | 2 |
| eGFR | 1.4 |
| anti-hypertensive drugs | 1.1 |
| Hcy | 1.1 |
| smoking | 1.4 |
| alcohol use | 1.3 |
| diabetes mellitus | 1.1 |
| lipid-lowering drugs | 1 |

| Step 2-The relationship between variables and HUA was examined one by one | | | | | |
| --- | --- | --- | --- | --- | --- |
| Covariates | β | exp(β) | 95%CI Low | 95%CI Up | *P*-value |
| Sex | -1.308 | 0.270 | 0.252 | 0.290 | <0.001 |
| Age | 0.006 | 1.006 | 1.002 | 1.010 | <0.001 |
| BMI | 0.055 | 1.057 | 1.047 | 1.067 | <0.001 |
| SBP | -0.007 | 0.994 | 0.992 | 0.995 | <0.001 |
| DBP | 0.006 | 1.006 | 1.003 | 1.009 | <0.001 |
| eGFR | -0.041 | 0.960 | 0.958 | 0.962 | <0.001 |
| anti-hypertensive drugs | 0.198 | 1.219 | 1.137 | 1.307 | <0.001 |
| Hcy | 0.038 | 1.039 | 1.035 | 1.043 | <0.001 |
| smoking | 0.611 | 1.841 | 1.707 | 1.987 | <0.001 |
| alcohol use | 0.806 | 2.240 | 2.064 | 2.430 | <0.001 |
| diabetes mellitus | 0.192 | 1.211 | 1.112 | 1.318 | <0.001 |
| lipid-lowering drugs | 0.197 | 1.218 | 1.109 | 1.159 | <0.001 |

| Selected variables |  |  |
| --- | --- | --- |
| Y | X | Selected variables |
| HUA | TC/HDL-C ratio | age, sex, BMI, SBP, DBP, current smoking, alcohol use, eGFR, Hcy, diabetes mellitus, anti-hypertensive drugs and lipid-lowering drugs. |

**Table S3. Screening for variables in the relationship between TG/HDL-C ratio and HUA**

| Step 1-Collinearity screening | |
| --- | --- |
|  | Step 1 (VIF) |
| **TG/HDL-C ratio** | 1.1 |
| Sex | 1.6 |
| Age | 1.9 |
| BMI | 1.2 |
| SBP | 1.7 |
| DBP | 2 |
| eGFR | 1.4 |
| anti-hypertensive drugs | 1.1 |
| Hcy | 1.1 |
| smoking | 1.4 |
| alcohol use | 1.3 |
| diabetes mellitus | 1.1 |
| lipid-lowering drugs | 1 |

| Step 2-The relationship between variables and HUA was examined one by one | | | | | |
| --- | --- | --- | --- | --- | --- |
| Covariates | β | exp(β) | 95%CI Low | 95%CI Up | *P*-value |
| Sex | -1.308 | 0.270 | 0.252 | 0.290 | <0.001 |
| Age | 0.006 | 1.006 | 1.002 | 1.010 | <0.001 |
| BMI | 0.055 | 1.057 | 1.047 | 1.067 | <0.001 |
| SBP | -0.007 | 0.994 | 0.992 | 0.995 | <0.001 |
| DBP | 0.006 | 1.006 | 1.003 | 1.009 | <0.001 |
| eGFR | -0.041 | 0.960 | 0.958 | 0.962 | <0.001 |
| anti-hypertensive drugs | 0.198 | 1.219 | 1.137 | 1.307 | <0.001 |
| Hcy | 0.038 | 1.039 | 1.035 | 1.043 | <0.001 |
| smoking | 0.611 | 1.841 | 1.707 | 1.987 | <0.001 |
| alcohol use | 0.806 | 2.240 | 2.064 | 2.430 | <0.001 |
| diabetes mellitus | 0.192 | 1.211 | 1.112 | 1.318 | <0.001 |
| lipid-lowering drugs | 0.196 | 1.207 | 1.121 | 1.312 | <0.001 |

| Selected variables |  |  |
| --- | --- | --- |
| Y | X | Selected variables |
| HUA | TG/HDL-C ratio | age, sex, BMI, SBP, DBP, current smoking, alcohol use, eGFR, Hcy, diabetes mellitus, anti-hypertensive drugs and lipid-lowering drugs. |

**Table S4. Screening for variables in the relationship between LDL-C/HDL-C ratio and HUA**

| Step 1-Collinearity screening | |
| --- | --- |
|  | Step 1 (VIF) |
| **LDL-C/HDL-C ratio** | 1.2 |
| Sex | 1.6 |
| Age | 1.9 |
| BMI | 1.2 |
| SBP | 1.7 |
| DBP | 2 |
| eGFR | 1.4 |
| anti-hypertensive drugs | 1.1 |
| Hcy | 1.2 |
| smoking | 1.4 |
| alcohol use | 1.3 |
| diabetes mellitus | 1 |
| lipid-lowering drugs | 1 |

| Step 2-The relationship between variables and HUA was examined one by one | | | | | |
| --- | --- | --- | --- | --- | --- |
| Covariates | β | exp(β) | 95%CI Low | 95%CI Up | *P*-value |
| Sex | -1.308 | 0.270 | 0.252 | 0.290 | <0.001 |
| Age | 0.006 | 1.006 | 1.002 | 1.010 | <0.001 |
| BMI | 0.055 | 1.057 | 1.047 | 1.067 | <0.001 |
| SBP | -0.007 | 0.994 | 0.992 | 0.995 | <0.001 |
| DBP | 0.006 | 1.006 | 1.003 | 1.009 | <0.001 |
| eGFR | -0.041 | 0.960 | 0.958 | 0.962 | <0.001 |
| anti-hypertensive drugs | 0.198 | 1.219 | 1.107 | 1.337 | <0.001 |
| Hcy | 0.038 | 1.039 | 1.035 | 1.043 | <0.001 |
| smoking | 0.611 | 1.841 | 1.707 | 1.987 | <0.001 |
| alcohol use | 0.806 | 2.240 | 2.064 | 2.430 | <0.001 |
| diabetes mellitus | 0.192 | 1.211 | 1.112 | 1.318 | <0.001 |
| lipid-lowering drugs | 0.065 | 1.017 | 1.136 | 1.167 | <0.001 |

| Selected variables |  |  |  |
| --- | --- | --- | --- |
| Y |  | X | Selected variables |
| HUA |  | LDL-C/HDL-C ratio | age, sex, BMI, SBP, DBP, current smoking, alcohol use, eGFR, Hcy, diabetes mellitus, anti-hypertensive drugs and lipid-lowering drugs. |

**Table S5. Screening for variables in the relationship between non-HDL-C and HUA**

| Step 1-Collinearity screening | |
| --- | --- |
|  | Step 1 (VIF) |
| **non-HDL-C** | 1.1 |
| Sex | 1.6 |
| Age | 1.8 |
| BMI | 1.2 |
| SBP | 1.7 |
| DBP | 2 |
| eGFR | 1.4 |
| anti-hypertensive drugs | 1.1 |
| Hcy | 1.2 |
| smoking | 1.4 |
| alcohol use | 1.3 |
| diabetes mellitus | 1.1 |
| lipid-lowering drugs | 1 |

| Step 2-The relationship between variables and HUA was examined one by one | | | | | |
| --- | --- | --- | --- | --- | --- |
| Covariates | β | exp(β) | 95%CI Low | 95%CI Up | *P*-value |
| Sex | -1.308 | 0.270 | 0.252 | 0.290 | <0.001 |
| Age | 0.006 | 1.006 | 1.002 | 1.010 | <0.001 |
| BMI | 0.055 | 1.057 | 1.047 | 1.067 | <0.001 |
| SBP | -0.007 | 0.994 | 0.992 | 0.995 | <0.001 |
| DBP | 0.006 | 1.006 | 1.003 | 1.009 | <0.001 |
| eGFR | -0.041 | 0.960 | 0.958 | 0.962 | <0.001 |
| anti-hypertensive drugs | 0.198 | 1.219 | 1.137 | 1.307 | <0.001 |
| Hcy | 0.038 | 1.039 | 1.035 | 1.043 | <0.001 |
| smoking | 0.611 | 1.841 | 1.707 | 1.987 | <0.001 |
| alcohol use | 0.806 | 2.240 | 2.064 | 2.430 | <0.001 |
| diabetes mellitus | 0.192 | 1.211 | 1.112 | 1.318 | <0.001 |
| lipid-lowering drugs | 0.177 | 1.208 | 1.115 | 1.317 | <0.001 |

| Selected variables |  |  |
| --- | --- | --- |
| Y | X | Selected variables |
| HUA | non-HDL-C | age, sex, BMI, SBP, DBP, current smoking, alcohol use, eGFR, Hcy, diabetes mellitus, anti-hypertensive drugs and lipid-lowering drugs. |

**Table S6** Goodness of fit assessment of regression models

|  | -2 log likelihood | Nagelkerke R^2 | Accurate percentage |
| --- | --- | --- | --- |
| TC/HDL-C ratio | 14896.243 | 0.335 | 72.6% |
| TG/HDL-C ratio | 14855.390 | 0.338 | 72.9% |
| LDL-C/HDL-C ratio | 14940.190 | 0.332 | 72.7% |
| non-HDL-C | 14852.549 | 0.338 | 72.7% |


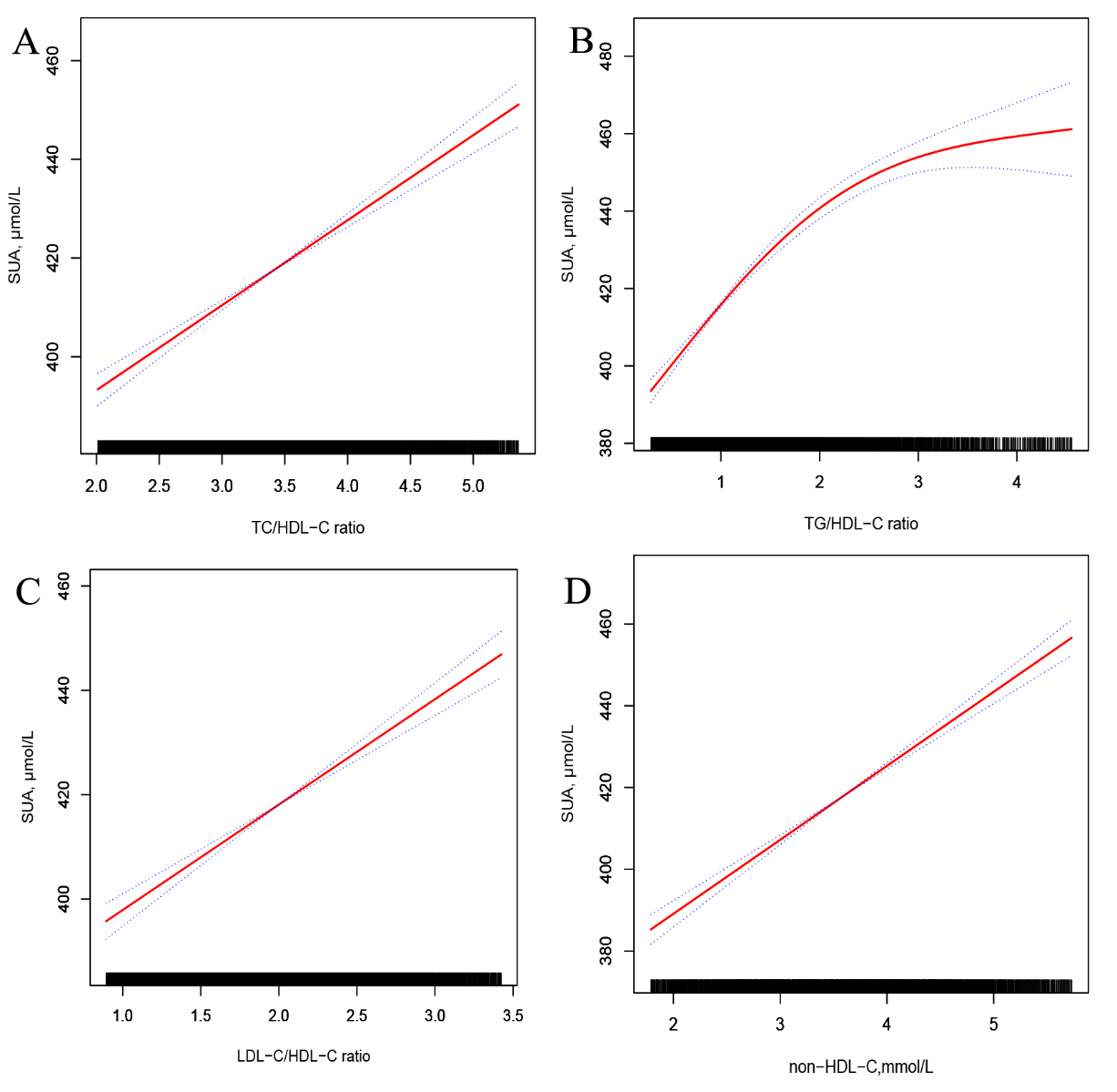


**Fig. S1**

| **Table S7** Each cut-off points of lipid profiles corresponding to HUA (420 μmol/L) | | | |  |
| --- | --- | --- | --- | --- |
| TC/HDL-C ratio | TG/HDL-C ratio | LDL-C/HDL-C ratio | non-HDL-C, mmol/L |  |
| 3.46 | 1.21 | 2.03 | 3.61 |  |

Adjusted for age, sex, BMI, SBP, DBP, current smoking, alcohol use, eGFR, Hcy, diabetes mellitus and anti-hypertensive drugs.

| **Table S8**. Association between four lipid profiles and HUA (Non-smoker=10,564) | | | | |
| --- | --- | --- | --- | --- |
|  | Crude model |  | Adjusted model |  |
| Variables | OR (95%CI) | *P* value | OR (95%CI) | *P* value |
| TC/HDL-C ratio | 1.44 (1.38, 1.51) | <0.001 | 1.40 (1.32, 1.48) | <0.001 |
| TG/HDL-C ratio | 1.26 (1.21, 1.30) | <0.001 | 1.26 (1.21, 1.31) | <0.001 |
| LDL-C/HDL-C ratio | 1.56 (1.47, 1.66) | <0.001 | 1.51 (1.39, 1.63) | <0.001 |
| non-HDL-C | 1.23 (1.18, 1.28) | <0.001 | 1.36 (1.29, 1.42) | <0.001 |
| Adjusted model: adjusted for age, sex, BMI, SBP, DBP, alcohol use, eGFR, Hcy, diabetes mellitus, anti-hypertensive drugs and lipid-lowering drugs.   \| **Table S9**. Association between four lipid profiles and HUA (Non-drinker=11,158) \| \| \| \| \| \| --- \| --- \| --- \| --- \| --- \| \|  \| Crude model \|  \| Adjusted model \|  \| \| Variables \| OR (95%CI) \| *P* value \| OR (95%CI) \| *P* value \| \| TC/HDL-C ratio \| 1.50 (1.43, 1.57) \| <0.001 \| 1.40 (1.33, 1.48) \| <0.001 \| \| TG/HDL-C ratio \| 1.29 (1.25, 1.34) \| <0.001 \| 1.28 (1.23, 1.33) \| <0.001 \| \| LDL-C/HDL-C ratio \| 1.65 (1.55, 1.75) \| <0.001 \| 1.50 (1.40, 1.61) \| <0.001 \| \| non-HDL-C \| 1.21 (1.17, 1.26) \| <0.001 \| 1.34 (1.28, 1.41) \| <0.001 \| \| Adjusted model: adjusted for age, sex, BMI, SBP, DBP, current smoking, eGFR, Hcy, diabetes mellitus, anti-hypertensive drugs and lipid-lowering drugs.   \| **Table S10**. Association between four lipid profiles and HUA (eGFR>30 ml/min/1.73m^2^=14,021) \| \| \| \| \| \| --- \| --- \| --- \| --- \| --- \| \|  \| Crude model \|  \| Adjusted model \|  \| \| Variables \| OR (95%CI) \| *P* value \| OR (95%CI) \| *P* value \| \| TC/HDL-C ratio \| 1.38 (1.32, 1.43) \| <0.001 \| 1.34 (1.28, 1.41) \| <0.001 \| \| TG/HDL-C ratio \| 1.26 (1.23, 1.30) \| <0.001 \| 1.27 (1.23, 1.32) \| <0.001 \| \| LDL-C/HDL-C ratio \| 1.46 (1.38, 1.53) \| <0.001 \| 1.40 (1.31, 1.49) \| <0.001 \| \| non-HDL-C \| 1.19 (1.15, 1.24) \| <0.001 \| 1.33 (1.28, 1.39) \| <0.001 \| \| Adjusted model: adjusted for age, sex, BMI, SBP, DBP, current smoking, alcohol use, eGFR, Hcy, diabetes mellitus, anti-hypertensive drugs and lipid-lowering drugs. \| \| \| \| \| \| \| \| \| \| | | | | |

| **Table S11**. Association between four lipid profiles and HUA (Non-diabetic=11,610) | | | | |
| --- | --- | --- | --- | --- |
|  | Crude model |  | Adjusted model |  |
| Variables | OR (95%CI) | *P* value | OR (95%CI) | *P* value |
| TC/HDL-C ratio | 1.35 (1.29, 1.41) | <0.001 | 1.33 (1.26, 1.40) | <0.001 |
| TG/HDL-C ratio | 1.27 (1.23, 1.32) | <0.001 | 1.30 (1.24, 1.35) | <0.001 |
| LDL-C/HDL-C ratio | 1.43 (1.35, 1.51) | <0.001 | 1.38 (1.29, 1.49) | <0.001 |
| non-HDL-C | 1.14 (1.10, 1.19) | <0.001 | 1.30 (1.24, 1.36) | <0.001 |
| Adjusted model: adjusted for age, sex, BMI, SBP, DBP, current smoking, alcohol use, eGFR, Hcy, anti-hypertensive drugs and lipid-lowering drugs. | | | | |

| **Table S12**. Association between four lipid profiles and HUA (Not taking lipid-lowering drugs=13,721) | | | | |
| --- | --- | --- | --- | --- |
|  | Crude model |  | Adjusted model |  |
| Variables | OR (95%CI) | *P* value | OR (95%CI) | *P* value |
| TC/HDL-C ratio | 1.39 (1.33, 1.44) | <0.001 | 1.36 (1.29, 1.43) | <0.001 |
| TG/HDL-C ratio | 1.27 (1.23, 1.31) | <0.001 | 1.28 (1.23, 1.33) | <0.001 |
| LDL-C/HDL-C ratio | 1.47 (1.40, 1.55) | <0.001 | 1.42 (1.33, 1.51) | <0.001 |
| non-HDL-C | 1.20 (1.16, 1.25) | <0.001 | 1.35 (1.30, 1.41) | <0.001 |
| Adjusted model: adjusted for age, sex, BMI, SBP, DBP, current smoking, alcohol use, eGFR, Hcy, diabetes mellitus and anti-hypertensive drugs. | | | | |

| **Table S13. Subgroup analysis of the relationship between lipid ratio and HUA in gender** | | |
| --- | --- | --- |
| Subgroup | Adjusted OR (95%CI) | *P* for interaction |
| **TC/HDL-C ratio** | |  |
| Gender |  | <0.001 |
| male | 1.24 (1.16, 1.32) |  |
| female | 1.47 (1.37, 1.57) |  |
| **TG/HDL-C ratio** | |  |
| Gender |  | 0.584 |
| male | 1.29 (1.21, 1.37) |  |
| female | 1.26 (1.21, 1.32) |  |
| **LDL-C/HDL-C ratio** | |  |
| Gender |  | <0.001 |
| male | 1.26 (1.16, 1.37) |  |
| female | 1.59 (1.45, 1.74) |  |
| **non-HDL-C** |  |  |
| Gender |  | 0.155 |
| male | 1.29 (1.22, 1.37) |  |
| female | 1.37 (1.30, 1.45) |  |

Adjusted for age, BMI, SBP, DBP, current smoking, alcohol use, eGFR, Hcy, diabetes mellitus, anti-hypertensive drugs and lipid-lowering drugs.

| **Table S14. Lipid levels in different genders** | | | |
| --- | --- | --- | --- |
| gender | male (n=6,716) | female (n=7,511) | *P* value |
| Age, years | 63.82 ± 9.79 | 63.80 ± 8.96 | 0.903 |
| TC | 4.94 ± 1.06 | 5.35 ± 1.13 | <0.001 |
| LDL-C | 2.84 ± 0.78 | 3.11 ± 0.82 | <0.001 |


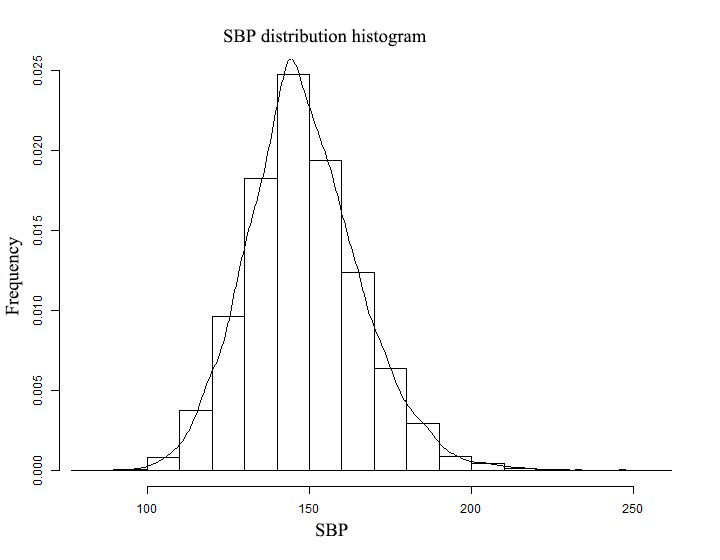
**Fig S2**

| **Table S15. SBP: Frequency distribution** | | | | |
| --- | --- | --- | --- | --- |
| Lower limit of grouping interval | Upper limit of grouping interval | Median of grouping interval | In-group frequency | Percentage |
| 80 | 90 | 85 | 4 | 0.0281 |
| 90 | 100 | 95 | 12 | 0.0843 |
| 100 | 110 | 105 | 114 | 0.8013 |
| 110 | 120 | 115 | 536 | 3.7675 |
| 120 | 130 | 125 | 1369 | 9.6225 |
| 130 | 140 | 135 | 2593 | 18.2259 |
| 140 | 150 | 145 | 3518 | 24.7276 |
| 150 | 160 | 155 | 2754 | 19.3576 |
| 160 | 170 | 165 | 1760 | 12.3708 |
| 170 | 180 | 175 | 908 | 6.3822 |
| 180 | 190 | 185 | 419 | 2.9451 |
| 190 | 200 | 195 | 129 | 0.9067 |
| 200 | 210 | 205 | 67 | 0.4709 |
| 210 | 220 | 215 | 23 | 0.1617 |
| 220 | 230 | 225 | 10 | 0.0703 |
| 230 | 240 | 235 | 4 | 0.0281 |
| 240 | 250 | 245 | 4 | 0.0281 |
| 250 | 260 | 255 | 3 | 0.0211 |


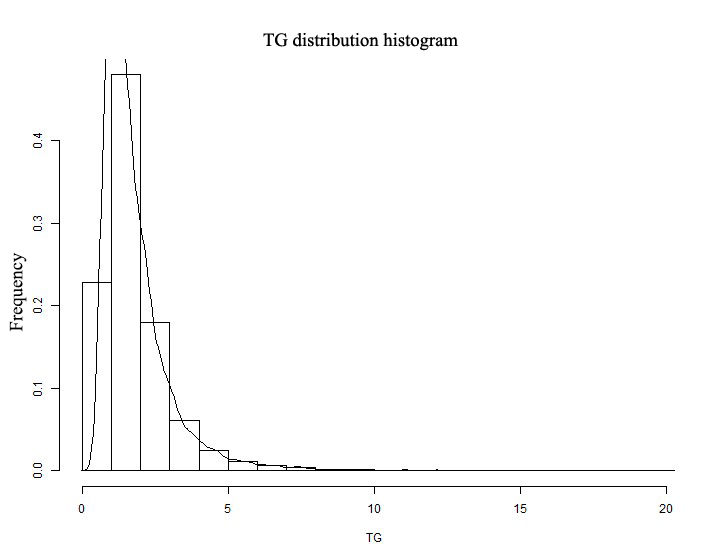
 **Fig S3**


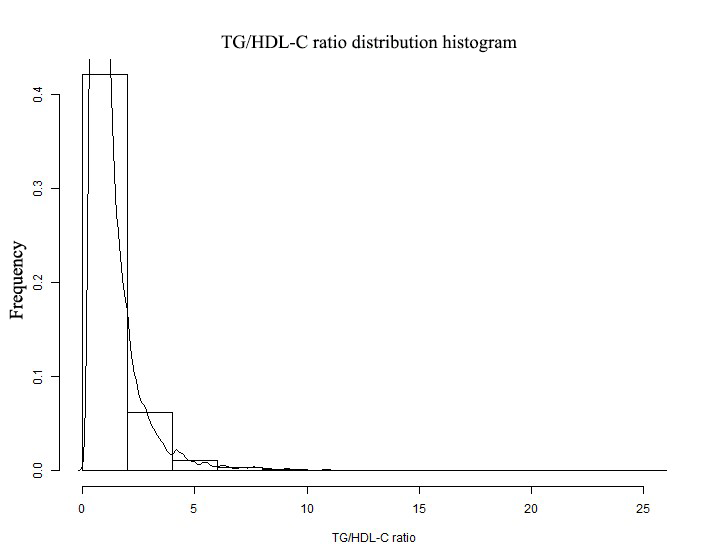
 **Fig S4**

| **Table S16. Antihypertensive and Lipid-lowering drugs** | |
| --- | --- |
| **Drugs** | N (%) |
| **Antihypertensive drugs*** | 9223 (64.84) |
| CCB | 5234 (36.79) |
| ACEI | 1558 (10.95) |
| ARB | 623 (4.38) |
| β-receptor blocker | 254 (1.79) |
| Diuretics | 756 (5.31) |
| others | 2004 (14.09) |
| **Lipid-lowering drugs** | 506 (3.56) |
| Atorvastatin | 141 (0.99) |
| Simvastatin | 260 (1.83) |
| Rosuvastatin | 90 (0.63) |
| Pravastatin | 15 (0.11) |

Abbreviations: CCB, calcium channel blockers; ACEI, angiotensin converting enzyme inhibitor; ARB, angiotensin receptor blockers

* Note: some patients have taken more than two antihypertensive drugs.
